# Yi-nao-jie-yu Prescription Exerts a Positive Effect on Neurogenesis by Regulating Notch Signals in the Hippocampus of Post-stroke Depression Rats

**DOI:** 10.3389/fpsyt.2018.00483

**Published:** 2018-10-16

**Authors:** Huiling Tian, Xiaoli Li, Qisheng Tang, Wen Zhang, Qingmeng Li, Xinyue Sun, Ruizhen Zhao, Chongyang Ma, Haipeng Liu, Yushan Gao, Fei Han

**Affiliations:** ^1^Department of Encephalopathy, The Third Affiliated Hospital, Beijing University of Chinese Medicine, Beijing, China; ^2^Research Institute, Beijing University of Chinese Medicine, Beijing, China

**Keywords:** post-stroke depression, Yi-nao-jie-yu prescription, notch signaling, neurogenesis, forced swim test, sucrose consumption test

## Abstract

Post-stroke depression (PSD) is one of the most frequent complications of stroke. The Yi-nao-jie-yu prescription (YNJYP) is an herbal prescription widely used as a therapeutic agent against PSD in traditional Chinese medicine. Disruption of adult neurogenesis has attracted attention as a potential cause of cognitive pathophysiology in neurological and psychiatric disorders. The Notch signaling pathway plays an important role in neurogenesis. This study investigated the effects of YNJYP on adult neurogenesis and explored its underlying molecular mechanism in a rat model of PSD that is established by middle cerebral artery occlusion and accompanied by chronic immobilization stress for 1 week. At 2, 4, and 8 weeks, depression-like behavior was evaluated by a forced swim test (FST) and sucrose consumption test (SCT). Neurogenesis was observed by double immunofluorescence staining. Notch signals were detected by real-time polymerase chain reaction. The results show that, at 4 weeks, the immobility time in the FST for rats in the PSD group increased and the sucrose preference in the SCT decreased compared with that in the stroke group. Therefore, YNJYP decreased the immobility time and increased the sucrose preference of the PSD rats. Further, PSD interfered with neurogenesis and decreased the differentiation toward neurons of newly born cells in the hippocampal dentate gyrus, and increased the differentiation toward astrocytes, effects that were reversed by YNJYP, particularly at 4 weeks. At 2 weeks, compared with the stroke group, expression of target gene *Hes5* mRNA transcripts in the PSD group decreased, but increased after treatment with YNJYP. At 4 weeks, compared with the stroke group, the expression of Notch receptor *Notch1* mRNA transcripts in the PSD group decreased, but also increased after treatment with YNJYP. Overall, this study indicated that disturbed nerve regeneration, including the increased numbers of astrocytes and decrease numbers of neurons, is a mechanism of PSD, and Notch signaling genes dynamically regulate neurogenesis. Moreover, YNJYP can relieve depressive behavior in PSD rats, and exerts a positive effect on neurogenesis by dynamically regulating the expression of Notch signaling genes.

## Introduction

Post-stroke depression is one of the most frequent complications of stroke, with an estimated prevalence as high as 80% (1). It often takes a chronic course, and is associated with increased morbidity and mortality, and a poorer functional outcome (2–4). Although risk factors, including metabolic factors, impairment of cognitive functions, and social factors, are associated with PSD (5), its pathophysiology remains unclear.

Recently, disruption of adult neurogenesis has attracted attention as a potential cause of cognitive pathophysiology in neurological and psychiatric disorders, such as depression, anxiety, schizophrenia, and bipolar disorder. Numerous studies have demonstrated that stress inhibits neurogenesis in the dentate gyrus (DG) of the hippocampus. Conversely, antidepressants are known to promote adult hippocampal neurogenesis (6). While the regulatory mechanism at the molecular level remains obscure, Notch signaling has been conserved throughout evolution, and plays a fundamental role in neuronal progenitor maintenance and subsequent control of differentiation (7, 8). Gaining a clearer understanding of pathogenesis is essential for the development of better treatments.

The Chinese herbal preparation known as Yi-nao-jie-yu prescription (YNJYP) has been shown to be effective for both body function recovery and the treatment of depression in patients with PSD. In animal studies, the brain damage in PSD model rats was greater than that in stroke model rats, and YNJYP was able to reverse this effect (9). In this study, we hypothesized that disruption of neurogenesis is a pathogenic mechanism of PSD, and that alteration of Notch signaling is the therapeutic mechanism of the beneficial effects of YNJYP at the molecular level. Dynamic changes in neurogenesis and fluctuations in Notch signals were observed in the hippocampus of PSD model rats, and the mechanism of YNJYP for the treatment of PSD was explored.

## Materials and methods

### Animals

Adult male Sprague-Dawley rats aged 7–9 weeks (300–320 g) were purchased from Vital River Laboratory Animal Technology (No. SCXK2012-0001). Five animals were housed per cage at 23 ± 3°C and a humidity of 45 ± 5%, under a 12-h light/dark cycle (lights on at 8:00 a.m., lights off at 8:00 p.m.), and received standard sterile food and water *ad libitum*. Animals were allowed to acclimatize for 1 week before the study. All procedures were approved and performed according to the guidelines of the Beijing University of Chinese Medicine Animal Care and Use Committee. Experimental protocols were approved by the Animal Experimentation Ethics Committee of Beijing University of Chinese Medicine. Efforts were made to minimize the number of animals used and the suffering of experimental animals.

### Combined middle cerebral artery occlusion and depression model

All surgeries, behavioral testing, and histological analysis were performed by a single investigator. Animals were numbered upon arrival at the animal facility, and randomly divided into a sham operation (Sham) group and a surgery group. Left middle cerebral artery occlusion (MCAO) was performed in the surgery group, using a modification of the technique described in our previous study (9). The animals were intraperitoneally anesthetized with sodium pentobarbital (50 mg/kg) for all surgical procedures. Subsequently, MCAO was performed using a monofilament suture with a rounded tip that was introduced into the internal carotid artery carefully and advanced approximately to the origin of the middle cerebral artery. The monofilament was left *in situ*, and withdrawn after 2 h for reperfusion. The temporalis muscle temperature was maintained at 37.0–37.5°C by surface heating until the rats recovered from anesthesia. After recovery from anesthesia, the neurological status of the rats was assessed according to a 5-point scale described by Longa et al. (10). A post-operative care plan was employed to help prevent weight loss and dehydration over the first week after surgery. This included the supply of paracetamol in drinking water (120 mg/kg), sub-cutaneous injections of saline (days 1–3), and provision of soft food. For the first week after surgery, animals were housed in a recovery room where they could be closely monitored. After the seventh day post-surgery, animals were returned to general holding rooms. Additional animals were allocated to longer recovery timepoints to allow for deaths and to ensure enough animals survived up to each timepoint. Data from animals which did not reach their designated endpoint was not included in the analysis.

Finally, a total of 159 rats were used in this study; of these, 27 rats were in the Sham group. The 108 survivors with neurological scores of 1–3 were randomly divided into four groups as follows: stroke group (n=27), PSD group (*n* = 27), fluoxetine hydrochloride (FXT) group (*n* = 27), and YNJYP group (*n* = 27), for further study. Rats were allowed 1 week to recover from the MCAO operation. To establish a PSD model, rats in PSD, FXT, and YNJYP groups were subjected to isolation housing in combination with chronic unexpected mild stress [behavioral restriction: immobilized on wooden shelves, as described by Chen et al. (11), for 2 h at any time of the day, consecutively for 1 week].

### YNJYP preparation and treatment

Granules of YNJYP were provided by the Pharmacy Department of the Third Affiliated Hospital of Beijing University of Chinese Medicine. Six different Chinese medical herbs were included in the YNJYP: 30 g Manyprickle *Acanthopanax* sp. root, 10 g *Radix curcumae*, 15 g Fructus *Schisandrae chinensis*, 10 g Fructus *Gardeniae* sp., 15 g *Salviae miltiorrhizae*, and 15 g *Rhizoma chuanxiong*. The granules were dissolved in 100 ml of distilled water and maintained at 4°C for further use. Rats in the sham, stroke, and PSD groups were gavaged with 10 ml/kg saline (0.9%). Rats in the FXT group were gavaged with 2.33 mgkg^−1^day^−1^ of FXT (0943A; Patheon France, Jiangsu, People's Republic of China), and the YNJYP rats were gavaged with 9.92 g/kg YNJYP once daily, following the MCAO operation. At 2, 4, or 8 weeks after the simulated stroke, rats were sacrificed after anesthetization for further study.

### BrdU injections

For the analysis of neurogenesis, rats (*n* = 3) in each group received an intraperitoneal injection of 50 mg/kg of the thymidine analog 5-bromo-2-deoxyuridine (BrdU) (b5002; Sigma, St. Louis, MO, USA,), freshly prepared at a concentration of 10 mg/ml dissolved in sterile 0.9% saline solution. The injections were started from the MCAO operation, twice a day for the first 3 days, then twice per week, and finally, once every 8 h for the last 2 days before the rats were sacrificed.

### Forced swim test

The procedure for the forced swim test (FST) was as described by Veena et al. (12). Rats were subjected to the FST at 2, 4, and 8 weeks. Briefly, animals were subjected to a trial during which they were forced to swim in a plastic bucket (60 cm high, 45 cm in diameter) filled with water (23–25°C) up to a height of 40 cm, so that the rats could not support themselves by touching the bottom with their feet. After 5 min, the rats were removed from the bucket, dried with a towel, and kept warm under a lamp in their home cages. The test was followed by water and food deprivation for 24 h. The analyst was blind to the groups, and recorded the swimming time, during which the rats were swimming along the wall or actively attempting to climb it. A rat was judged as immobile whenever it remained floating passively in the water and made only the movements necessary to keep its nose or head above the water. Rats were counted as mobile if they moved their forepaws or supported themselves by pressing their paws against the wall of the cylinder. After the test, dry towels were used to keep the rats warm and dry them gently.

### Sucrose consumption test

The sucrose consumption test (SCT) was conducted 2, 4, and 8 weeks after the simulated stroke. Rats were tested for sucrose consumption as described previously (12). Animals were housed individually throughout the test duration, and after water deprivation for 24 h, were presented simultaneously with two bottles in the home cage, one containing a 1% sucrose solution and the other containing standard drinking water, for 60 min. The volumes of water and sucrose-water intake were measured. To prevent a preference for position from affecting the results, the locations of the two bottles (right/left) were switched during this period. The amount of liquid remaining in each bottle was measured at the end of the testing period. The sucrose preference score was expressed as a percentage of total fluid intake.

### Double immunofluorescence staining

Brains of the three rats in each group that received BrdU injections were fixed in paraformaldehyde after cardiac perfusion. After 24 h, 30% sucrose was added, and after the brains sank to the bottom of the bottle, they were embedded in optimal cutting temperature compound, frozen, and sectioned. Proteinase K was added at 37°C after the brain sections were washed three times with phosphate-buffered saline for 5 min each. Then, 30 min later, the following primary antibodies were added: BrdU (ab115874, mouse, 1:150; Abcam, Cambridge, MA, USA) and nestin (ab92391, rabbit, 1:250; Abcam), BrdU and neuronal nuclear antigen (NeuN) (ab177487, rabbit, 1:1200; Abcam), or BrdU and glial fibrillary acidic protein (GFAP) (16825-1-AP, rabbit, 1:1200; Proteintech, Chicago, IL, USA). After incubation overnight at 4°C, the secondary antibodies tetramethylrhodamine isothiocyanate (ZF-0313, goat anti-rat IgG, 1:200; ZSGB-Bio, Beijing, China) and fluorescein isothiocyanate (ZF-0311, goat anti-rabbit IgG, 1:200; ZSGB-Bio) were added. Following incubation for 2 h at room temperature, 80 μL 4′,6-diamidino-2-phenylindole (bw-d0010; GeneBio, Beijing, China) was added to the sections. A laser scanning confocal microscope (FV1000, Olympus Corporation, Tokyo, Japan) was used to view the hippocampal DG.

### Real-time PCR

The left hippocampus (*n* = 6) was dissected and frozen at −80°C. Total RNA from the hippocampus was isolated using Trizol reagent, according to the manufacturer's protocol (DP405-02; Tiangen Biotech Co., Ltd., Beijing, China). The gene primers were as follows:

**Table:** 

**Gene**	**Forward primer**	**Reverse primer**
*Notch1*	5′-TGGATGCCGCTGACCTACG-3′	5′-TGGATGCCGCTGACCTACG-3′
*Jagged1*	5′-TTAGTAAACGGGATGGGAACAGC-3′	5′-AAGCAACAGACCCAAGCCACT-3′
*Hes1*	5′-TTGAGCCAACTGAAAACACTGATT- 3′	5′-GTGCTTCACTGTCATTTCCAGAAT-3′
*Hes5*	5′-GATGCTCAGTCCC AAGGAGAAAA-3′	5′-CCACGAGTAACCCT CGCTGTAGT-3′
*GAPDH*	5′-CCTTCCGTGTTCCTACCCC-3′	5′-GCCCAGGATGCCCTTTAGTG-3′

The basic protocol for real-time polymerase chain reaction (PCR) was an initial denaturation at 95°C for 30 s, followed by 40 cycles of amplification. For cDNA amplification, cycles consisted of 95°C for 10 s and 60°C for 60 s. The final elongation step was from 60 to 99°C, at a rate of 0.05°C/s. The SYBR green signal was then detected using a real-time PCR machine (ABI7500; Applied Biosystems, Waltham, MA, USA). The PCR products were analyzed by gel electrophoresis and melting curve analysis to confirm specific amplifications. The mRNA expression levels were normalized to those of *GAPDH*. Transcript levels were quantified using the 2-ΔΔCt-value method.

### Statistical analyses

Data were expressed as means±standard deviation, and analyzed using SPSS 20.0 statistical software (IBM, Armonk, NY, USA). Differences between groups were tested using one-way analysis of variance followed by the *post hoc* Fisher's least significant difference test or Kruskal–Wallis test followed by Dunn's test if the data were not normally distributed. Threshold for statistical significance was set to *P* < 0.05.

## Results

### Effects of YNJYP on the depressive behavior of PSD rats

**Figure 1:**
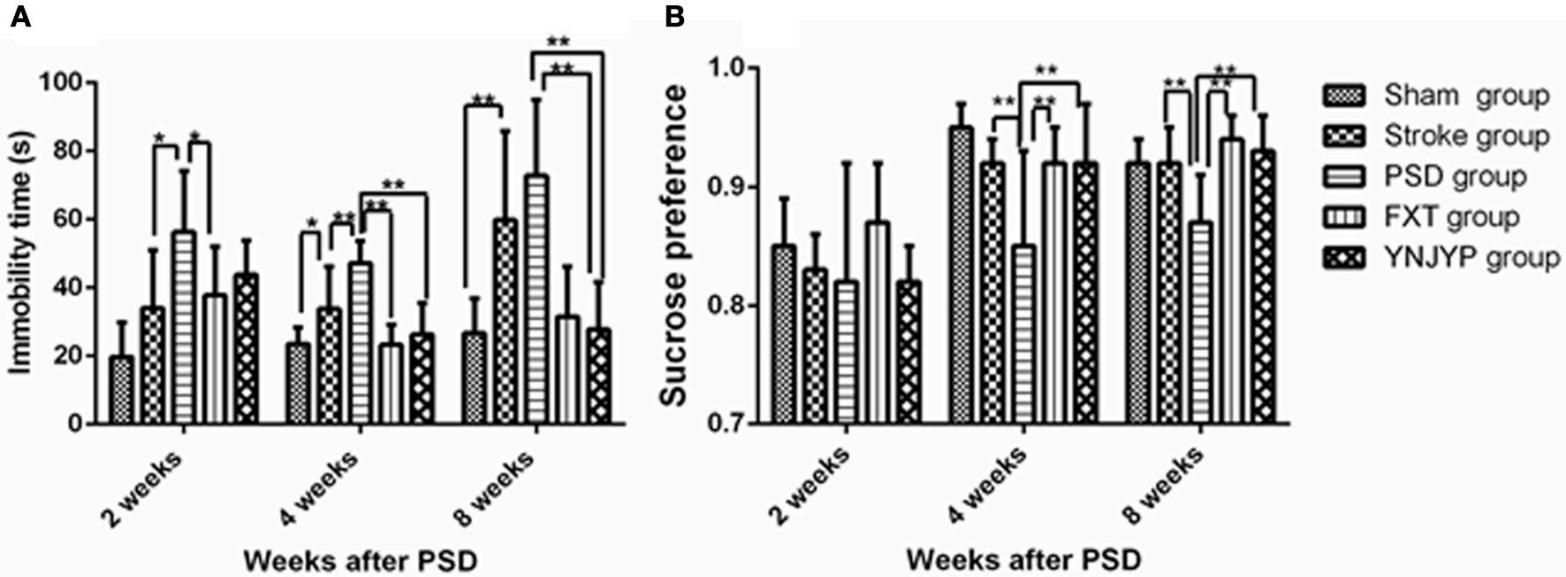
**(A)** Effect of treatments on the immobility time of PSD rats in the forced swim test. **(B)** Effect of treatments on the sucrose preference of PSD rats in the sucrose consumption test. Data are presented as mean ± standard error of the mean. *n* = 6; *P < 0.05, **P < 0.01. Sham group, sham operation group; PSD group, post-stroke depression group; FXT group, Fluoxetine hydrochloride capsules group; YNJYP group, Yi-nao-jie-yu prescription group.

Depression-like behavior was evaluated by FST and SCT. The immobility time in FST was used to assess the degree of despair in rats and the sucrose preference in the SCT of rats was used to reflect the rat's desire for good things. One way analysis of variance showed a significant difference in the immobility time (*n* = 6; 2 weeks: *F* = 3.973, *P* = 0.012; 4 weeks: *F* = 9.054, *P* < 0.001; 8 weeks: *F* = 8.085, *P* < 0.001; Figure 1A) and sucrose preference (*n* = 6; 2 weeks: *F* = 12.198, *P* = 0.431; 4 weeks: *F* = 11.875, *P* = 0.039; 8 weeks: *F* = 5.409, *P* = 0.003; Figure 1B) in the 5 groups. At 4 weeks, the immobility time in the FST for rats in the PSD group increased compared with that in the stroke group, and the difference was statistically significant (*P* < 0.01). Interestingly, YNJYP decreased the immobility time of the PSD rats, and at 4 and 8 weeks, the differences were statistically significant (*P* < 0.01 for both, Figure 1A). The sucrose preference in the SCT of rats in the PSD group decreased compared with that of rats in the stroke group, and at 4 and 8 weeks, and the differences were statistically significant (*P* < 0.01 for both). However, YNJYP increased the sucrose preference of the PSD rats at 4, and 8 weeks, and the differences were statistically significant (*P* < 0.01 for both, Figure 1B).

### Neurogenesis

#### Relationship between BrdU labeling and nestin immunoreactivity

**Figure 2:**
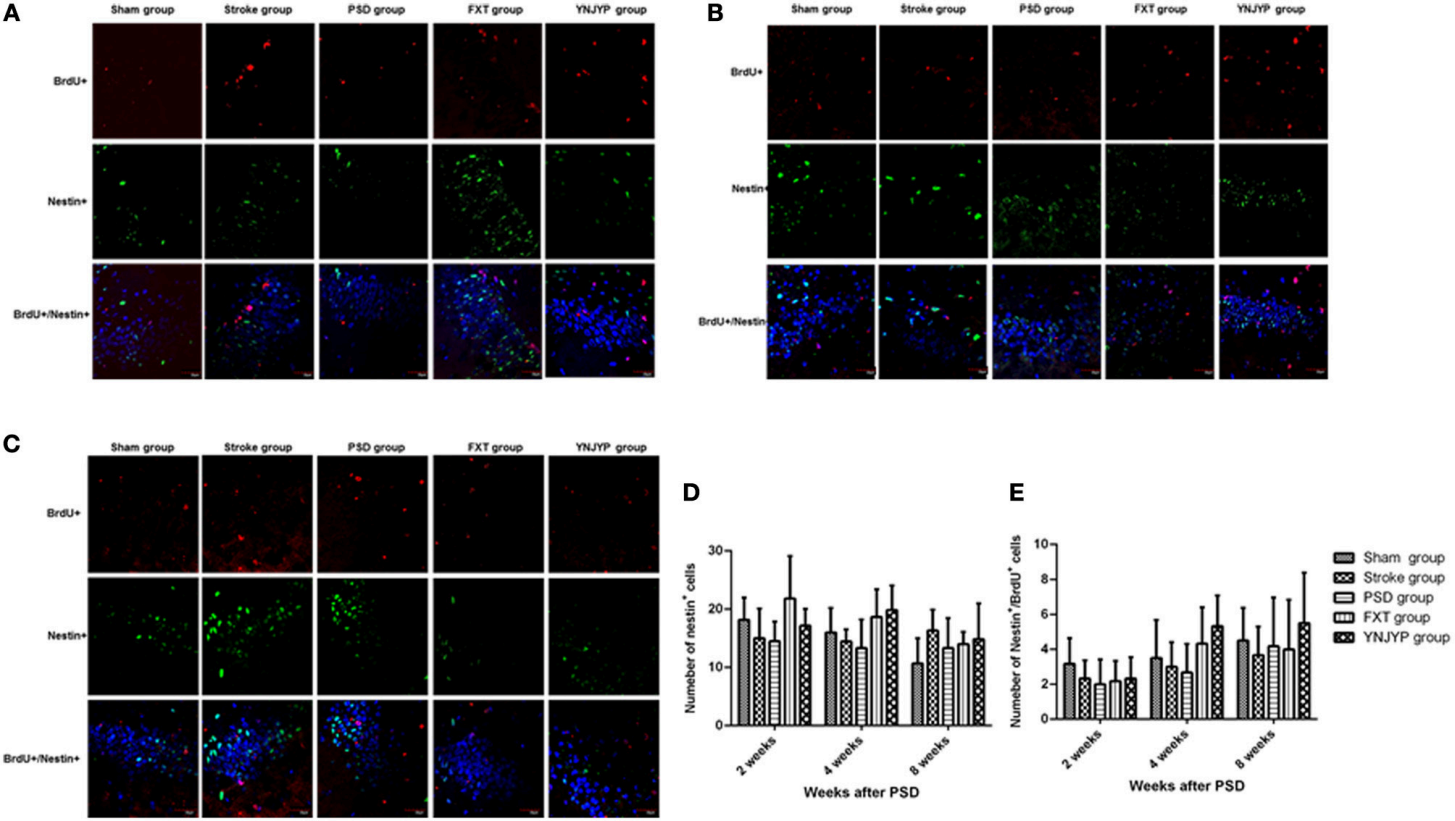
Effects of treatments on neural stem cell proliferation in PSD rats at 2, 4, and 8 weeks post-stroke. To observe the proliferation of neural stem cells (NSCs), newly formed cells were labeled with BrdU (red) and NSCs were labeled with nestin (green) at 2, 4, and 8 weeks post-stroke. Cell nuclei were labeled with 4′,6-diamidino-2-phenylindole (blue). **(A)** At 2 weeks post-stroke. **(B)** At 4 weeks post-stroke. **(C)** At 8 weeks post-stroke. Scale bar = 30 μm. **(D)** The number of nestin-positive cells at the observed dentate gyrus zone (at 2 weeks: *F* = 2.274, *P* = 0.090; at 4 weeks: *F* = 2.563, *P* = 0.063; at 4 weeks: *F* = 1.314, *P* = 0.292). **(E)** The number of cells double-positive for nestin and BrdU appeared at the dentate gyrus (at 2 weeks: *F* = 0.754, *P* = 0.565; at 4 weeks: *F* = 2.086, *P* = 0.113; at 8 weeks: *F* = 0.488, *P* = 0.745). BrdU, 5-bromo-2-deoxyuridine; PSD, post-stroke depression; FXT, fluoxetine hydrochloride capsules; YNJYP, Yi-nao-jie-yu prescription.

To observe the proliferation of neural stem cells (NSCs), we performed double-labeling at 2, 4, and 8 weeks with antibodies against BrdU (red), a marker for DNA replication in newly formed cells (13), and nestin (green), a neural progenitor-specific marker (14). Figures 2A–C shows that, at 2, 4, and 8 weeks, rats in the stroke group showed an increase in the number of BrdU-labeled (red) newly formed cells and nestin-labeled (green) NSCs in the DG, compared with the sham group. Nestin-positive or BrdU-positive cells could be observed in the PSD, FXT, and YNJYP groups, but only a few cells were positive for both nestin and BrdU. Figure 2D shows that the numbers of nestin-positive cells at the observed DG zone of rats in the five groups at 2, 4, and 8 weeks were not statistically significantly different (*F* = 2.274, *P* = 0.090; *F* = 2.563, *P* = 0.063; and *F* = 1.314, *P* = 0.292, respectively). Figure 2E shows that the numbers of cells double-positive for nestin and BrdU at the observed DG zone of rats in the five groups at 2, 4, and 8 weeks were not statistically significantly different (*F* = 0.754, *P* = 0.565; *F* = 2.086, *P* = 0.113; and *F* = 0.488, *P* = 0.745, respectively).

#### Relationship between BrdU labeling and NeuN immunoreactivity

**Figure 3:**
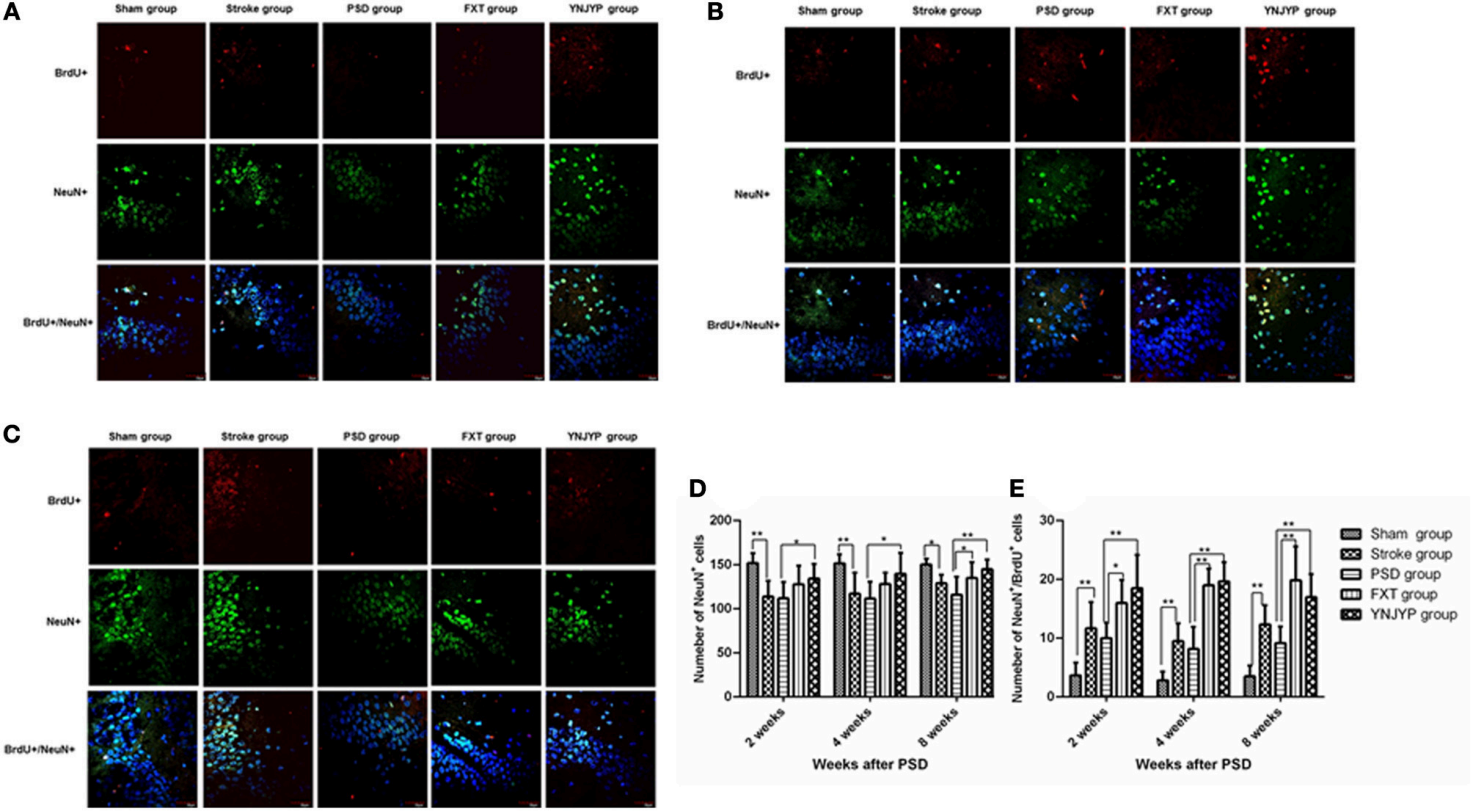
Effects of treatments on differentiation of newly formed cells toward neurons in PSD rats at 2, 4, and 8 weeks post-stroke. To observe the differentiation of newly formed cells toward neurons, recently divided cells were labeled with BrdU (red) and neurons were labeled with NeuN (green) at 2, 4, and 8 weeks. Cell nuclei were labeled with 4′,6-diamidino-2-phenylindole (blue). **(A)** At 2 weeks post-stroke. **(B)** At 4 weeks post-stroke. **(C)** At 8 weeks post-stroke. Scale bar = 30 μm. **(D)** The number of NeuN-positive cells at the observed dentate gyrus zone (at 2 weeks: *F* = 5.294, *P* = 0.003; at 4 weeks: *F* = 4.543, *P* = 0.007, at 8 weeks: *F* = 5.543, *P* = 0.002). **(E)** The number of cells double-positive for NeuN and BrdU at the observed dentate gyrus zone (at 2 weeks: *F* = 12.539, *P* = 0.001; at 4 weeks: *F* = 36.158, *P* < 0.001, at 8 weeks: *F* = 17.668, *P* < 0.001). *n* = 6; *P < 0.05, **P < 0.01. BrdU, 5-bromo-2-deoxyuridine; PSD, post-stroke depression; FXT, fluoxetine hydrochloride capsules; YNJYP, Yi-nao-jie-yu prescription.

To observe the differentiation of newly formed cells toward neurons, we performed double-labeling with antibodies against BrdU (red) and NeuN (green), a neuron-specific protein (15). Figures 3A–C shows that only a few cells double-positive for BrdU and NeuN were observed in rats in the sham, stroke, and PSD groups, but after treatment with FXT or YNJYP more BrdU-positive cells appeared near the DG, and these cells were also NeuN-positive at 2, 4, and 8 weeks. At 2 and 4 weeks, newly formed neurons appeared outside the granule cell layer, and had migrated into the granule cell layer at 8 weeks. Figure 3D shows that, compared with the sham group, fewer NeuN-positive cells appeared at the observed DG zone of rats in the stroke group at 2, 4, and 8 weeks, and this difference was statistically significant (*P* < 0.01, *P* < 0.01, and *P* < 0.05, respectively). More NeuN-positive cells appeared in rats in that YNJYP group than in rats in the PSD group, and the difference was statistically significant (*P* < 0.05, *P* < 0.05, and *P* < 0.01, respectively). Figure 3E shows that, compared with the sham group, more cells double-positive for NeuN and BrdU appeared at the DG of rats in the stroke group at 2, 4, and 8 weeks, and the difference was statistically significant (*P* < 0.01, *P* < 0.01, and *P* < 0.01, respectively). Further, more cells double-positive for NeuN and BrdU appeared in rats in the YNJYP group than in rats in the PSD group, and the difference was statistically significant (*P* < 0.01, *P* < 0.01, and *P* < 0.01, respectively). These results suggested that more newly formed neurons appeared at the DG after MCAO operation, and YNJYP could increase the number of newly formed neurons at the DG of PSD rats. However, not only the total number of neurons but also the number of newly formed neurons in PSD rats was not statistically significantly different from that of stroke rats. This suggested that the decrease in the number of neurons may be not the key factor for the depressive behavior of PSD rats.

#### Relationship between BrdU labeling and GFAP immunoreactivity

**Figure 4:**
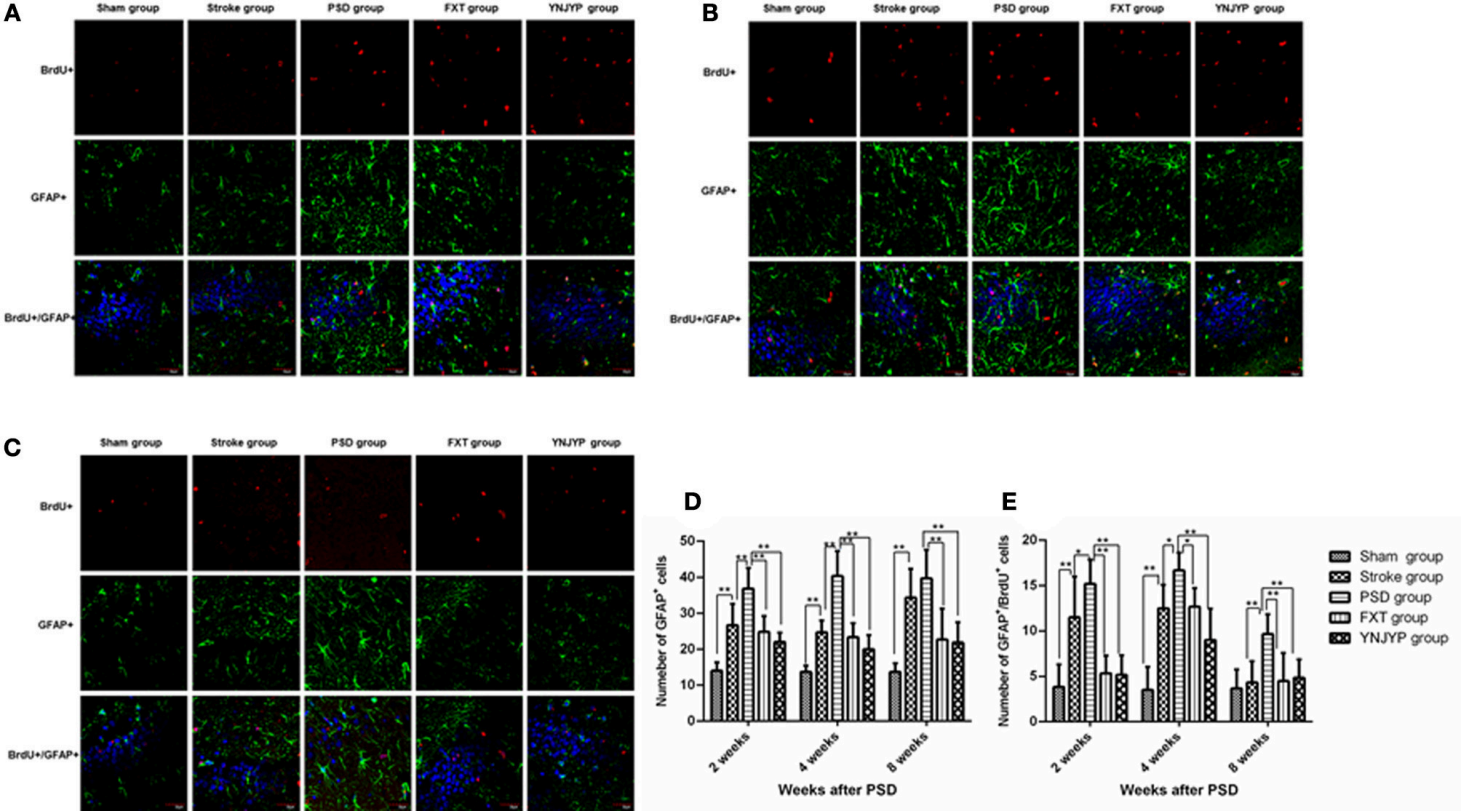
Effects of treatments on differentiation of newly formed cells toward astrocytes in PSD rats at 2, 4, and 8 weeks post-stroke. To observe the differentiation of newly formed cells toward astrocytes, newly formed cells were labeled with BrdU (red) and astrocytes were labeled with GFAP (green) at 2, 4, and 8 weeks. Cell nuclei were labeled with 4′,6-diamidino-2-phenylindole (blue). **(A)** At 2 weeks post-stroke. **(B)** At 4 weeks post-stroke. **(C)** At 8 weeks post-stroke. Scale bar = 30 μm. **(D)** The number of GFAP-positive cells at the observed dentate gyrus zone at 2, 4, and 8 weeks (at 2 weeks: *F* = 20.579, *P* < 0.001; at 4 weeks: *F* = 31.673, *P* < 0.001, at 8 weeks: *F* = 13.733, *P* < 0.001). **(E)** The number of cells double-positive for GFAP and BrdU at the observed dentate gyrus zone (at 2 weeks: *F* = 17.182, *P* < 0.001; at 4 weeks: *F* = 22.852, *P* < 0.001; at 8 weeks: *F* = 6.274, *P* = 0.001). *n* = 6; *P < 0.05, *P < 0.01. BrdU, 5-bromo-2-deoxyuridine; PSD, post-stroke depression; FXT, fluoxetine hydrochloride capsules; YNJYP, Yi-nao-jie-yu prescription.

To observe the differentiation of newly formed cells toward astrocytes, we performed double-labeling with antibodies against BrdU (red) and GFAP (green), an astrocyte-specific marker (16). Figures 4A–C shows that more GFAP-positive cells and cells double-positive for GFAP and BrdU appeared at the observed DG zone of the PSD rats than that of rats in the stroke group at 2, 4, and 8 weeks. However, fewer GFAP-positive cells and cells double-positive for GFAP and BrdU appeared at the observed DG zone of rats in the FXT or YNJYP group. At 2 weeks, in rats in the PSD group, astrocytes appeared outside the granule cell layer, and had migrated into the granule cell layer at 4 and 8 weeks. Figure 4D shows that the PSD rats showed a statistically significant increase in the number of GFAP positive cells at the observed DG zone at 2 and 4 weeks compared with rats in the stroke group (*P* < 0.01 for both). Both FXT and YNJYP decreased the number of astrocytes at the observed DG zone at 2, 4, and 8 weeks (*P* < 0.01, *P* < 0.01, and *P* < 0.01, respectively). Figure 4E shows that the PSD rats showed a statistically significant increase in the number of cells double-positive for GFAP and BrdU at the observed DG zone at 2 and 4 weeks compared with rats in the stroke group (*P* < 0.05, *P* < 0.05, and *P* < 0.01, respectively). Both FXT and YNJYP decreased the number of cells double-positive for GFAP and BrdU at the observed DG zone at 2, 4, and 8 weeks (*P* < 0.01, *P* < 0.01, and *P* < 0.01, respectively). This suggested that the increase in the number of newly formed astrocytes may be the key factor for the depressive behavior of PSD rats.

### Notch signaling pathway

Real-time PCR was used to measure *Notch1, Jagged1, Hes1*, and *Hes5* mRNA transcript levels in the hippocampus. Standard curves were generated for the *Notch1, Jagged1, Hes1, Hes5*, and *Gapdh* genes. Melting curve analysis confirmed no primer dimers in the PCR products.

#### Notch1 mRNA transcript expression in the hippocampus

**Figure 5:**
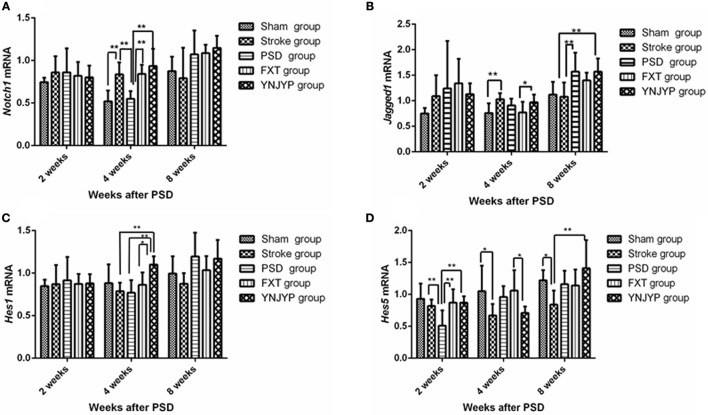
Effects of treatments on the expression levels of *Notch1, Jagged1, Hes1*, and *Hes5* mRNA transcripts in the hippocampus of PSD rats at 2, 4, and 8 weeks post-stroke. Real-time polymerase chain reaction was performed to investigate changes in the expression of *Notch1, Jagged1, Hes1*, and *Hes5* mRNA transcripts in PSD rats and the effects of YNJYP on them (*n* = 6 per group); *P < 0.05, **P < 0.01. **(A)** The expression of Notch1 mRNA in rats at 2, 4, and 8 weeks post-stroke (*n* = 6; 2 weeks: *F* = 0.413, *P* = 0.797; 4 weeks: *F* = 10.950, *P* < 0.001; 8 weeks: *F* = 12.006, *P* = 0.071). **(B)** The expression of Jagged1 mRNA in rats at 2, 4, and 8 weeks post-stroke (*n* = 6; 2 weeks: *F* = 1.154, *P* = 0.355; 4 weeks: *F* = 3.149, *P* = 0.032; 8 weeks: *F* = 4.582, *P* = 0.007). **(C)** The expression of Hes1 mRNA in rats at 2, 4, and 8 weeks post-stroke (*n* = 6; 2 weeks: *F* = 0.113, *P* = 0.977; 4 weeks: *F* = 4.688, *P* = 0.006; 8 weeks: *F* = 2.500, *P* = 0.068). **(D)** The expression of Hes5 mRNA of rats at 2, 4, and 8 weeks post-stroke (*n* = 6; 2 weeks: *F* = 4.646, *P* = 0.006; 4 weeks: *F* = 3.132, *P* = 0.032; 8 weeks: *F* = 3.358, *P* = 0.025). PSD, post-stroke depression; FXT, fluoxetine hydrochloride capsules; YNJYP, Yi-nao-jie-yu prescription.

Figure 5A shows the *Notch1* mRNA transcript expression in the hippocampus at 2, 4, and 8 weeks (*n* = 6; 2 weeks: *F* = 0.413, *P* = 0.797; 4 weeks: *F* = 10.950, *P* < 0.001; 8 weeks: *F* = 12.006, *P* = 0.071). At 4 weeks, the expression of *Notch1* mRNA transcripts for rats in the PSD group was lower than that for rats in the stroke group (*P* < 0.01), but significantly increased after treatment with FXT or YNJYP (*P* < 0.01). There were no significant differences between the YNJYP and FXT groups (*P* > 0.05). At 2 or 8 weeks, levels of *Notch1* mRNA transcripts of rats in the five groups were not statistically significantly different (*P* > 0.05).

#### Jagged1 mRNA transcript expression in the hippocampus

Figure 5B shows the *Jagged1* mRNA transcript expression in the hippocampus at 2, 4, and 8 weeks (*n* = 6; 2 weeks: *F* = 1.154, *P* = 0.355; 4 weeks: *F* = 3.149, *P* = 0.032; 8 weeks: *F* = 4.582, *P* = 0.007). At 2 weeks, the levels of *Jagged1* mRNA transcripts of rats in the five groups were not statistically significantly different (*P* > 0.05). At 4 weeks, compared with the sham group, the expression of *Jagged1* mRNA transcripts of rats in the stroke group were higher (*P* < 0.01). *Jagged1* mRNA transcript levels in the YNJYP group were higher than those in the FXT group (*P* < 0.05). At 8 weeks, the expression of *Jagged1* mRNA transcripts in the PSD group was higher than that in the stroke group (*P* < 0.01). There were no statistically significant differences between the YNJYP and PSD groups (*P* > 0.05). These data show that YNJYP was unable to reduce the expression of *Jagged1* mRNA transcripts in the PSD group at 8 weeks.

#### Hes1 mRNA transcript expression in the hippocampus

Figure 5C shows the *Hes1* mRNA transcript expression in the hippocampus at 2, 4, and 8 weeks (*n* = 6; 2 weeks: *F* = 0.113, *P* = 0.977; 4 weeks: *F* = 4.688, *P* = 0.006; 8 weeks: *F* = 2.500, *P* = 0.068). At 4 weeks, the expression levels of *Hes1* mRNA transcripts of rats in the stroke and PSD groups were similar (*P* > 0.05). After treatment by YNJYP, levels of *Hes1* mRNA transcript expression in the PSD rats increased (*P* < 0.01). At 2 or 8 weeks, levels of *Hes1* mRNA transcripts of rats in the five groups were similar, and showed no statistically significant differences (*P* > 0.05).

#### Hes5 mRNA transcript expression in the hippocampus

Figure 5D shows the *Hes1* mRNA transcript expression in the hippocampus at 2, 4, and 8 weeks (*n* = 6; 2 weeks: *F* = 4.646, *P* = 0.006; 4 weeks: *F* = 3.132, *P* = 0.032; 8 weeks: *F* = 3.358, *P* = 0.025). At 2 weeks, levels of *Hes5* mRNA transcripts of rats in the PSD group were significantly lower than those in the stroke group (*P* < 0.01). After treatment by FXT or YNJYP, the expression levels of *Hes5* mRNA transcript of rats in the PSD group increased significantly (*P* < 0.01). At 4 weeks, *Hes5* mRNA transcript levels in the YNJYP group were lower than those of the FXT group (*P* < 0.05). At 8 weeks, levels of *Hes5* mRNA transcripts of rats in the five groups were not statistically significantly different (*P* > 0.05).

## Discussion

In this study, MCAO was used as a rat model of stroke, and isolation housing combined with chronic immobilization stress was used as a model of post-stroke depression. Compared with the stroke group, PSD rats showed increased immobility time and decreased consumption of sucrose water at 4 weeks. This difference was statistically significant. Therefore, the PSD model used in our study appears to be valid, and at 4 weeks, the PSD-related changes were observed.

According to traditional Chinese medicine, PSD pathogenesis involves kidney deficiency, liver-Qi stagnation, and blood stasis (17). Based on these concepts, YNJYP was created to reinforce the kidneys and regulate the liver-Qi, as well as reduce phlegm and promote blood circulation. It has been proven to be effective for both body function recovery and the antidepressant treatment of patients with PSD. In this study, the effect of YNJYP was evaluated in comparison with FXT, and it proved effective against PSD (18) at 2, 4, and 8 weeks. Our findings demonstrated that YNJYP significantly reversed the depressive behavior of PSD rats in the FST and SCT. However, the therapeutic mechanism underlying the activity of YNJYP remains unclear. Therefore, we explored the mechanism of YNJYP activity by studying neurogenesis and Notch signaling.

Neurogenesis includes the proliferation of NSCs, differentiation (mainly toward neurons and astrocytes), and functional integration of newly formed cells. It is accepted that the proliferation of NSCs and differentiation of the newly formed cells into neurons can contribute to the reversal of depressive behavior and stress-induced cognitive dysfunction. Some newly formed cells that migrate into the granule cell layer are critical for the restoration of function after injury (13). Astrocytes are the most numerous and versatile glial cells in the brain, but some of their functions remain the subject of debate. Some studies have indicated that they are able to regulate neurogenesis in the hippocampus (19), and that they guide the growth and integration of the newly formed neurons (20). Other studies have indicated that excessive differentiation toward astrocytes or hypertrophy results in impaired neurogenesis (21, 22). Neurogenesis in the hippocampus is particularly important in cognitive, affective, and reproductive behaviors, while dysfunctional neurogenic patterns are likely to be involved in mood and psychiatric disorders (23). Stress and depression contribute to decreased neurogenesis, and antidepressant treatment has been shown to ameliorate depression-like behavior and increase neurogenesis (24). 12 found that stress significantly decreased the proliferation and survival of progenitor cells in the hippocampus, which was partially restored following oxotremorine treatment. Apple et al. (23) also found that antidepressant treatment following stroke, which increases neurogenesis, enhanced the proliferation of NSCs and improved their migration toward sites of brain damage.

To understand how YNJYP protected the brain in our present study, neurogenesis in the DG of the hippocampus was observed at 2, 4, and 8 weeks by double immunofluorescence staining. Our observations showed that neurogenesis did occur in the hippocampus, and stroke acted to increase it, which is consistent with previous findings (25, 26). Neurogenesis decreased in the PSD rats compared with the stroke rats, while the depressive behavior of the PSD rats in the FST and SCT was aggravated. We observed that nestin-positive cells or BrdU-positive cells appeared separately, while there were only a few cells double-positive for nestin and BrdU at any of the three timepoints. This observation indicates that, at 2, 4, and 8 weeks post-stroke, the proliferation of NSCs occurred at a low level. At each timepoint, there were a few newly formed neurons (cells double-positive for BrdU and NeuN) in the PSD rats, and many newly formed astrocytes (cells double-positive for BrdU and GFAP), whereas greater numbers of newly formed cells and fewer newly formed astrocytes appeared after treatment with YNJYP. Many astrocytes of rats in the PSD group migrated into the granule cell layer, which may impair the synapses and hinder neurogenesis (22). After treatment with YNJYP, more newly formed neurons migrated to the granule cell layer. At 4 weeks, this phenomenon was much more obvious than at 2 or 8 weeks.

Therefore, the changes observed in our experiment indicated that neurogenesis did occur in the hippocampus. However, PSD reduced neurogenesis in the stroke rats by preventing newly formed cells from differentiating toward neurons and increasing their likelihood of becoming astrocytes. The excessive differentiation toward astrocytes may be the key factor for the depression-like behavior. Interestingly, YNJYP alleviated depressive behavior and dynamically reversed the process of neurogenesis. It was at 4 weeks that YNJYP exerted its most positive effects on neurogenesis.

An important issue raised by this study is the nature of the mechanism at the molecular level. Notch signaling plays an important role during adult neurogenesis. The Notch receptor is activated on binding to the membrane-bound Delta or Serrate ligand present on an adjacent cell. This interaction triggers cleavage of Notch to release a cytoplasmic fragment that enters the nucleus and interacts with the DNA-binding protein, CBF/RBP-J, Suppressor of Hairless, LAG-1, which leads to the transcription of target genes such as *Hairy* and *Enhancer-of-split* (27). Androutsellis-Theotokis et al. (28) found that activation of Notch signaling promotes the growth of nerves and plays a key role in the self-repair process after nerve injury. However, other reports were not consistent with this conclusion, indicating instead that Notch signaling can induce neuronal cell death (29). As previously reported, Notch1 is the most important transmembrane Notch receptor and is required for the maintenance of NSCs in the adult hippocampus (30). Jagged1 is the canonical membrane-bound ligand of Notch signaling, and conditional inactivation of Jagged1 during adult neurogenesis depletes the NSC population and ultimately hinders neurogenesis (31). The transcription factors Hairy/Enhancer-of-split (named *Hes* in mammals) are major downstream targets of Notch signaling. They are essential to regulate neurogenesis, although their roles remain unclear. Some studies indicate that they suppress the transcription of precursor genes, resulting in the inhibition of neuronal differentiation (32). Other studies have reported that Hes1 and Hes5 are essential to promote differentiation (7, 8).

In the present study, we observed fluctuations in *Notch1, Jagged1, Hes1*, and *Hes5* mRNA transcript levels, and found that, at 2 weeks, compared with the stroke group, expression of *Hes5* in the PSD group was first decreased, but then increased after treatment by FXT or YNJYP, whereas expression levels of the receptor *Notch1* and ligand *Jagged1* were not significantly different among the five groups. Thus, these results support the conclusion that not only Notch signaling, but other signals also participate in the regulation of neurogenesis. At 4 weeks, compared with the stroke group, the expression of *Notch1* and *Hes1* mRNA transcripts in the PSD group decreased, and again increased after treatment with FXT or YNJYP. At 8 weeks, compared with the stroke group, the expression of *Jagged1* mRNA transcripts in the PSD group increased and remained at a high level, even after treatment with FXT or YNJYP. The present study suggests that YNJYP exerts diverse effects on Notch signals at different timepoints, and that other factors also participate in the Notch signaling pathway (33–35).

In summary, this current investigation indicates that YNJYP can alleviate depressive behavior, and exerts a positive effect on neurogenesis by increasing neurogenesis, promoting differentiation toward neurons, and inhibiting differentiation toward astrocytes. At 4 weeks, the effect of YNJYP on neurogenesis was maximum. The beneficial effects of YNJYP treatment may be mediated by the activation of the Notch signaling pathway.

This study identified dynamic effects of YNJYP on adult neurogenesis. We mechanistically explored its effects on Notch signals, both the major Notch receptor and ligand as well as downstream targets of Notch signals, at three timepoints, and found dynamic changes in the levels of these molecules. Understanding the regulatory mechanisms during development may have crucial implications for developing repair therapies for PSD treatment (7). The contributions of other signaling pathways and inhibitors of signal pathways involved in the neuroprotective effects of YNJYP are the subjects of ongoing studies.

## Author contributions

HT contributed to the interpretation of results and writing of manuscript; XL and QT contributed to the study design and interpretation of results; WZ and YG analyzed the data; QL, HL and XS conducted the experiments; CM, RZ, and FH reviewed and approved the manuscript.

### Conflict of interest statement

The authors declare that the research was conducted in the absence of any commercial or financial relationships that could be construed as a potential conflict of interest.

## Abbreviations

BrdU: 5-bromo-2-deoxyuridine
DG: dentate gyrus
FST: forced swim test
FXT: fluoxetine hydrochloride
GFAP: glial fibrillary acidic protein
MCAO: middle cerebral artery occlusion
NeuN: neuronal nuclear antigen
NSC: neural stem cell
PSD: post-stroke depression
SCT: sucrose consumption test
YNJYP: Yi-nao-jie-yu prescription.
